# Quantitative Assessment of Three-Dimensional Choroidal Vascularity and Choriocapillaris Flow Signal Voids in Myopic Patients Using SS-OCTA

**DOI:** 10.3390/diagnostics11111948

**Published:** 2021-10-20

**Authors:** Amin Xu, Gongpeng Sun, Chaoye Duan, Zhen Chen, Changzheng Chen

**Affiliations:** Eye Center, Renmin Hospital of Wuhan University, Wuhan 430060, China; xam110708gy@163.com (A.X.); sungongpeng@whu.edu.cn (G.S.); Carterduan@hotmail.com (C.D.)

**Keywords:** myopia, three-dimensional choroidal vascularity index, choriocapillaris, flow signal voids, swept-source optical coherence tomography angiography

## Abstract

Purpose: To compare the choroidal vascularity of large- and middle-sized choroidal vessels and choriocapillaris (CC) perfusion in patients with different degrees of myopia using swept-source optical coherence tomography angiography (SS-OCTA). Methods: One hundred and thirteen people with myopia were enrolled. SS-OCTA was performed to analyze the choroidal vascularity and CC perfusion. Three-dimensional (3D) choroidal vascularity index (CVI) and choroidal luminal volumes (LV) were obtained by artificial intelligence segmentation of the choroidal lumen in Volume OCT images. CC perfusion was assessed by flow signal voids (FSVs). Results: In the macular, multiple linear regression model showed that choroidal thickness (CT), total choroidal volume, LV, and choroidal stromal volume were negatively correlated with axis length (AL), respectively (all *p* < 0.001). Three dimensional CVI was negatively associated with AL (*p* < 0.05). FSV% was positively correlated with age only (*p* < 0.001). Additionally, after adjustment for age and AL, FSV% had a significant negative correlation with CT (*p* < 0.05). Conclusion: Choroidal vascularity decreases gradually with increasing severity of myopia. The decrease of CC blood perfusion was related to a higher severity of myopia and the thinning of choroid.

## 1. Introduction

Myopia has become a global public health problem [1]. It has been reported that by 2050, there will be 4.758 billion people with myopia, accounting for 49.8% of the world population, and 938 million people with high myopia, accounting for 9.8% of the world population [2]. High myopia can result in irreversible vision loss such as retinal detachment, choroidal neovascularization, and macular atrophy [3]. Therefore, it is urgent to better understand the risk factors for developing myopia.

Previous studies have demonstrated^†^ that the retina becomes thinner with increasing axial length (AL), leading to the degeneration of fundus and visual impairment [4]. Besides the changes in the retina, the choroid also alternates in the development of myopia. The choroid is the main tissue that provides oxygen and nutrition and removes metabolites and wastes for the outer retina, and it may directly participate in the fundus degeneration and development of pathological myopia [5]. Recently, Zhang et al. also proposed that decrease in choroidal blood perfusion is responsible for the development of human myopia [6]. Additionally, choroidal vascularity and choriocapillaris (CC) blood perfusion may be lower in the more myopic eyes of anisomyopic adults [7]. Sonoda et al. observed that choroidal luminal areas decrease with longer AL [8]. 

However, these studies focused on line scans or single measurement points through the macula, which may not be sufficient to reveal changes in the overall state of choroidal vessels. The choroidal vasculature should be evaluated in three-dimensional (3D) rather than from a two-dimensional scan [9,10]. Swept-source optical coherence tomography angiography (SS-OCTA) with a map of 3D choroidal vascularity index (CVI) can provide detailed and accurate choroidal images [9]. Carefully studying the 3D CVI in different regions will help elucidate the choroidal vascular changes that accompany the development of myopia.

This study aimed to investigate alterations in choroidal vascularity of large- and middle-sized choroidal vessels and CC blood perfusion in 3D using SS-OCTA, and to explore the relationship among them in a cohort of eyes with different severities of myopia.

## 2. Methods

### 2.1. Participants

This prospective cross-sectional observational study was approved by the Clinical Research Ethics Committee of Renmin Hospital of Wuhan University (Approval Number WDRY2020-K234), and conducted according to the Declaration of Helsinki. Each subject provided written informed consent prior to the examination.

One hundred and thirteen participants with myopia were recruited from Renmin Hospital of Wuhan University from December 2020 to April 2021. All participants underwent best-corrected visual acuity, slit-lamp examination, intraocular pressure (IOP) measurement, optometry, AL measurement (IOLMaster 500, Carl Zeiss Meditec AG, Oberkochen, Germany), and SS-OCT. According to spherical equivalent refraction (SER), the participants were divided into three groups: low myopia (SER ≤ −0.5 to > −3.00 D), moderate myopia (SER ≤ −3.00 to > −6.00 D), and high myopia (SER ≤ −6.00 D) [11]. All subjects were free of ocular and systemic disease and had best-corrected Snellen visual acuity of 20/20 or better in each eye. Exclusion criteria included history of ocular surgery, smoking, hypertension, diabetes, or other systemic diseases that may affect the choroid. All participants underwent examinations between 10:00 and 12:00 every day to control for diurnal variations of choroidal structure [12]. Three consecutive scans were performed, and the clearest image was selected for analysis. Only the right eye of each subject was included for analyses.

### 2.2. Image Acquisition and Analysis

**Figure 1:**
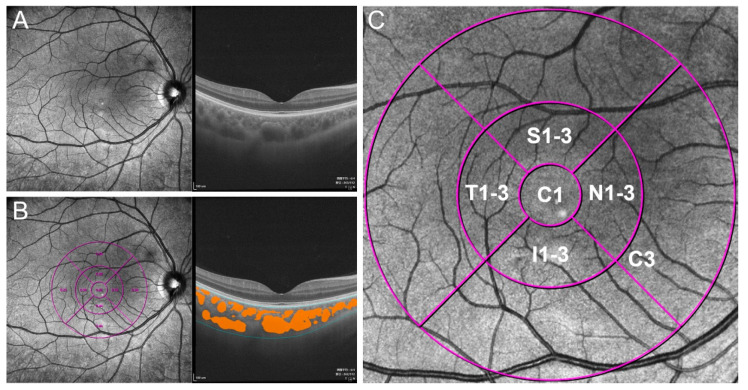
Illustration of extraction of choroidal lumen and calculation of three-dimensional choroidal vascularity index (3D CVI). (**A**) Raw SS-OCT images. (**B**) Measurement of choroidal thickness and lumen. The superior and inferior boundary of the choroid (blue line) and choroidal vascular lumen (orange area) were identified by deep learning. (**C**) Magnified non-infrared image with 3-mm ETDRS grid. C1, central fovea; C3, parafovea; I1-3, inferior parafovea; T1-3, temporal parafovea; S1-3, superior parafovea; N1-3, nasal parafovea. C3 was divided into I1-3, T1-3, S1-3, and N1-3.

The SS-OCT (VG100; SVision Imaging, Ltd., Luoyang, China) system used contained an SS laser with a central wavelength of approximately 1050 nm and a scan rate of 100,000 A-scans per second. The maximum axial and estimated lateral resolutions in tissue were approximately 5 µm and 15 µm, respectively. The scan depth was 3 mm. The volume data of choroidal vascularity and choroidal thickness (CT) were acquired with a raster scan protocol of 1024 × 1024 B-scans, which covered an area of 6 × 6 mm centered on the macular fovea. Eye-motion artifacts during and between scans were minimized by using built-in eye-tracking and follow-up mode of the device based on an integrated confocal scanning laser ophthalmoscope. The choroid in the SS-OCT images was defined as the volume from the outer boundary of the retinal pigment epithelium-Bruch membrane complex to the choroid-sclera junction (Figure 1A,B). The 3D CVI was defined as the ratio of the choroidal luminal volume (LV) to the total choroidal volume (TCV), which reflects the choroidal vascular density of Sattler’s layer and Haller’s layer. CT, 3D CVI, and LV values are presented for early treatment diabetic retinopathy study (ETDRS) grids of 6 × 6 mm using deep learning with the instrument software (Figure 1B). Choroidal stromal volume (SV) is defined as TCV minus LV. According to the ETDRS grids, the macular area was divided into three concentric circles with diameters of 1 mm (central fovea, C1), 3 mm (parafovea, C3), and 6 mm (perifovea). Given the presence of certain defects and artifacts at the scan edges of the images, only the images within 3 mm were selected. The parafoveal circle was further divided into superior (S1-3), inferior (I1-3), temporal (T1-3), and nasal (N1-3) quadrants (Figure 1C). 

**Figure 2:**
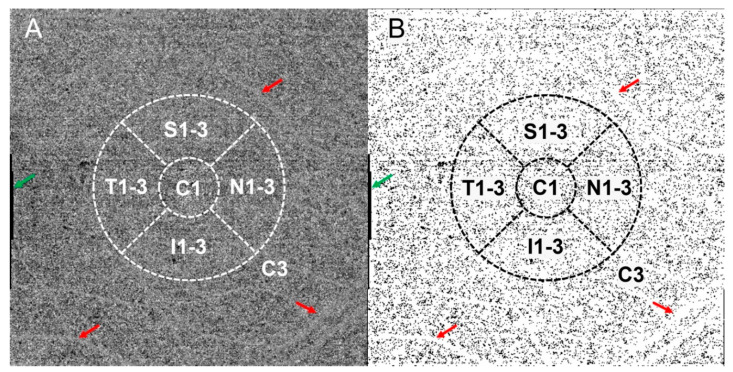
Illustration of flow signal voids (FSV) acquisition and calculation. (**A**) Raw en face choriocapillaris (CC) images with 3-mm ETDRS grid. (**B**) Binary image of CC with 3-mm ETDRS grid. The black particles in the image are the extracted FSVs. Artifacts caused by motion (green arrow) and retinal vascular projection (red arrow) were observed around the CC.

The slab of OCTA blood flow signal from the Bruch membrane to 20 μm below was layered using the machine’s built-in software into choriocapillaris images (Figure 2A). The FSV%, defined as the percentage of low signal area of the CC image detected by the binarization method to the total measured area, was quantified using ImageJ (version 1.53c, http://fiji.sc/; accessed on 26 June 2020). The raw images were converted into eight-bit images and the flow signal voids (FSVs) were identified using Auto Local Threshold tool with the Sauvola method [13]. The Analyze Particles tool was used to calculate FSV%. Quantitative analysis of CC was carried out according to the same regional division in 3D CVI analysis. Due to the obvious projection of large retinal vessels above the CC images beyond Region C3 and the presence of certain defects and artifacts in the surrounding areas of the images, only images within 3 mm were selected for analysis (Figure 2B).

### 2.3. Statistical Analysis

The demographic and clinical characteristics of the three different myopic groups were analyzed. Due to the small sample size, most parameters were not normally distributed, except for AL and all 3D CVI (by the Shapiro-Wilk test and histogram). All the data are presented as mean ± standard deviation or median (interquartile range). The Kruskal-Wallis H test or one-way ANOVA test followed by Bonferroni was performed to identify differences in demographic and clinical characteristics between the three myopia groups. The sex difference was determined by the Chi-square test. Multivariate linear regression was performed to investigate the correlation between choroidal parameters, age and AL, and to evaluate the correlations between choroidal parameters after adjustment for age and AL. Spearman correlation was used to evaluate the correlations between choroidal parameters. Statistical analysis was performed using SPSS 20.0 (IBM, Armonk, NY, USA). *p* < 0.05 was considered statistically significant.

## 3. Results

### 3.1. Study Population

A total of 113 people with myopia were recruited in this study. The mean age of enrolled subjects was 29.43 ± 10.18 years (range: 19–62 years), which included 66 women (58.41%). They were divided into three groups according to SER. The demographic characteristics of the three groups are presented in Table 1. 

### 3.2. Global Analysis of Differences in Choroidal Vascularity and Choriocapillaris

The choroidal parameters within the range of 3 mm centered on the macula were used to analyze the global differences in choroidal parameters between the three myopia groups (see Table S1, which provides global analysis of choroidal parameters between different myopia groups). In Regions C3, CT, TCV, and SV were significantly different between any two groups (all *p* < 0.05). The 3D CVI was significantly lower in high myopia group compared with low and moderate myopia groups (both *p* < 0.01), while no significant difference was observed between low and moderate groups (*p* = 1.00). Additionally, FSV% was significantly different between low and high myopia groups only (*p* = 0.036).

### 3.3. Topographical Analysis of Differences in Choroidal Vascularity and Choriocapillaris

**Figure 3:**
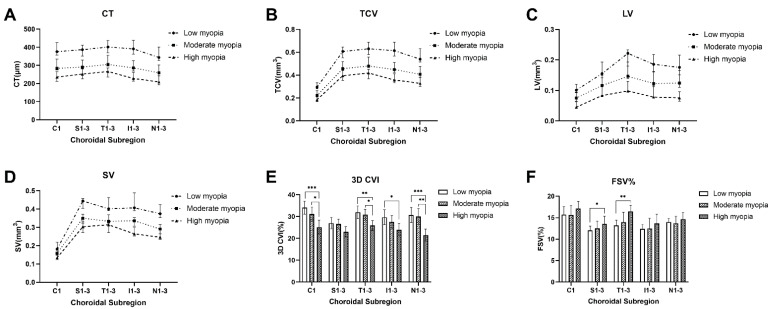
Topographic analysis of choroidal thickness (CT), total choroidal volume (TCV), luminal volume (LV), stromal volume (SV), three-dimensional choroidal vascularity index (3D CVI), and flow signal voids% (FSV%) between myopia groups in subregions (**A**–**F**). CT, TCV, LV, SV, and FSV% are expressed as medians and 95% confidence intervals, and 3D CVI is expressed as mean ± standard deviation. In all subregions, CT, TCV, LV, and SV were significantly lower in high and moderate myopia groups than in low myopia group (all *p* < 0.01, determined by the Kruskal-Wallis H test). In all subregions, the 3D CVI did not differ between low and moderate myopia groups (all *p* > 0.05, determined by the one-way ANOVA test), while there were differences between the other two comparison groups. In Region T1-3 and Region S1-3, FSV% was significantly higher in high myopia group compared with low myopia group (*p* = 0.001 and *p* = 0.023, respectively, determined by Kruskal-Wallis H test). * *p* < 0.05, ** *p* < 0.01, and *** *p* < 0.001 indicate significant differences between myopia groups.

The comparison of choroidal parameters between these groups is summarized in Figure 3. Overall, CT, TCV, LV, and SV differed between people with different degrees of myopia, although there were no significant differences in some subregions. In all subregions, the 3D CVI did not differ between low and moderate myopia groups (all *p* > 0.05), while there were differences between the other two comparison groups. In Region T1-3 and Region S1-3, FSV% was significantly higher in high myopia group compared with low myopia group (*p* = 0.001 and *p* = 0.023, respectively).

### 3.4. Correlation between Choroidal Parameters and Age and AL

Next, we used multivariate linear regression to control for potentially confounding variables to explore the correlation between choroidal parameters and AL and age in Regions C1 and C3 (Table 2). The multiple linear regression model showed that CT, TCV, LV, and SV were negatively correlated with AL respectively in both regions (all *p* < 0.001). In addition, in Region C3, 3D CVI was negatively associated with age and AL respectively (both *p* < 0.05), whereas in Region C1, 3D CVI was only negatively associated with AL (*p* < 0.001). FSV% remained positively correlated with age only in both regions (both *p* < 0.001).

### 3.5. Correlation between Choroidal Parameters

Spearman correlation coefficients between 3D CVI and other choroidal parameters in Regions C1 and C3 are summarized (see Table S2, which provides correlation analysis between 3D CVI and other choroidal parameters). There was a significant positive correlation between 3D CVI and CT, TCV, LV, and SV respectively in both regions (all *p* < 0.001). Conversely, a significant negative correlation between 3D CVI and FSV% was observed in both regions (both *p* < 0.01). Multiple linear regression showed that FSV% correlated negatively with 3D CVI% in both Region C1 and Region C3 (standardized coefficient = −0.282, *p* = 0.003 and standardized coefficient = −0.339, *p* < 0.001, respectively). Additionally, after adjustment for age and AL, FSV% had a significant negative correlation with CT in both Regions 1 and 3 (standardized coefficient = −0.216, *p* = 0.039 and standardized coefficient = −0.392, *p* < 0.001, respectively). 

## 4. Discussion

This study quantitatively analyzed the alterations of choroidal vascularity and CC blood perfusion in 3D in the eyes of patients from three groups with different refractive states by using SS-OCTA. We observed that parameters describing choroidal vascularity, assessed by CT, TCV, LV, and SV and 3D CVI, were lower in choroidal regions in high myopia group compared with low and moderate myopia groups, whereas the FSV% in CC was significantly higher in high myopia group compared with low myopia group in Regions C3, T1-3, and S1-3. Moreover, there were correlations between most of the choroidal parameters and the severity of myopia, including age and AL. The alterations of choroidal vasculature negatively correlated with AL, while FSV% was positively correlated with age only.

The choroid is composed of stroma and blood vessels, containing three layers: the CC, Sattler’s layer of medium-sized vessels, and Haller’s layer of large-sized vessels [14]. Previous studies mainly evaluated choroidal changes by CT [15,16]. However, CT has great variability due to the influence of age, IOP, and other physiological factors [17]. CVI is more reproducible, so it can be better used to evaluate changes in choroidal structure [17]. Several previous studies have reported alterations of CVI values in myopic eyes [7,18]. However, these studies only evaluated the choroid using two-dimensional images. To comprehensively evaluate the choroidal circulation, we assessed choroidal vascularity, CC FSV%, and 3D CVI to provide a more accurate assessment of the vasculature than possible with two-dimensional CVI. 

In our study, 3D CVI decreased gradually with increasing severity of myopia, showing a significant trend but no significant differences in pairwise comparisons between adjacent groups. This discrepancy may be related to the small and uneven sample size of each group, or the 3D CVI of the adjacent groups may be changed by an amount below the detection threshold of the instrument. Zhou et al. [19] showed that technology may be limited due to the small changes in choroidal parameters, and this technical limitation may be reduced with the advent of new devices and updated algorithms. Previous studies have shown that choroidal thinning is related to accelerating ocular growth or the development of myopia [16,20]. Consistently, our results showed that CT was negatively correlated with AL. Additionally, TCV, LV, SV, and 3D CVI were all negatively correlated with AL in Regions C1 and C3 in our study. Li et al. [21] also reported negative correlations of luminal area with AL in low to moderately myopic children. Similarly, we observed that LV was lower in high and moderate myopia groups compared with low myopia group. In addition, we found that 3D CVI was positively correlated with CT in myopic eyes. Recent study also showed that choroidal thinning is mainly due to the loss of medium and large vessels [22]. Therefore, we propose that choroidal blood volume decreases with increasing AL or choroidal thinning in myopic eyes.

Some studies have reported increased FSV% or flow deficit in the CC of eyes with high myopia [7,23]. We speculate that progressive FSV may be an important feature in the development of pathological myopia. However, although FSV% had a generally increasing trend with increasing myopia, no significant differences were found between any regions of any two adjacent groups in our study. Furthermore, FSV% was found to be significantly higher in high myopia group than in low myopia group only in Zones T1-3 and S1-3. This regional difference was also very intriguing. We speculate that it may be related to small sample size or large age span or that old participants were included in low myopia group, given that FSV% was positively correlated with age. Previous studies have confirmed that CC flow deficits in the macula show progressive worsening with age [24]. Su et al. [25] proposed that, although the defect of CC blood flow seems worse in more myopic eyes, it cannot be ruled out that CC abnormalities may be caused by simple expansion or extension of the CC, rather than by CC atrophy. However, no matter what mechanism, prospective longitudinal studies will be required to further clarify the pathophysiology and progression of CC impairment in the development of myopia.

The CC is supplied and drained by Sattler’s layer of medium-sized vessels and Haller’s layer of large-sized vessels, so changes in blood flow in these two layers may cause alterations to CC perfusion [22]. Interestingly, by analyzing the relationship between FSV%, 3D-CVI, and CT, we found that FSV% was negatively correlated with 3D CVI% and CT, while CT was positively correlated with 3D CVI in Regions 1 and 3 among the different myopia groups. These results indicate that choroidal vascularity and CC perfusion decreased significantly with choroid thinning during myopic development. However, further longitudinal studies are needed to confirm whether it is the decrease of choroidal vascularity and CC perfusion that leads to myopia. Recent studies showed that choroidal blood flow decreases during induction of myopia [6,7], while another animal study also found that decreased choroidal blood flow can induce or aggravate myopia [26]. Therefore, the specific causal relationship remains unclear.

In the current study, we found region-specific differences in parameters describing choroidal vascularity. For instance, changes in 3D CVI were larger in the nasal and macular regions in high compared to low myopia groups, which may be related to differences in the decrease of stromal and vascular components in each region, as suggested previously [27]. Compared with 3D CVI, CT showed higher regional variability across the retina. CT generally is the thinnest in the nasal region, as reported previously [28,29]. It was reported that the choroid is the thickest within the perifovea, thins substantially towards the periphery, and exhibits maximum peripheral thinning in the nasal region [28]. These results indicate that the macular region is more sensitive to the development of myopia. This may be because the macular area contains the highest concentration of photoreceptors and engages in high levels of metabolism. The thickness of the nasal choroid was thinner than that in other regions, which may be related to the separation of the choroidal vascular bed by watershed zones in the choroid of the optic disc [30].

There are several limitations that should be considered. First, our sample size is relatively small, which could affect the homogeneity of the sample. Meanwhile, we did not conduct a long-term longitudinal study. Second, the large age span of our samples may lead to an inaccurate assessment of the relationship between CC and myopia. Third, we did not rule out factors that affect the choroid when we included the population, such as caffeine. 

Our study took advantage of several novel methodologies. First, the 3D volume scans with enhanced scan depth can improve image quality, which can be used to evaluate the choroidal blood flow comprehensively. Second, very few studies assessed CC and choroidal vascularity via so many parameters in the same patient population. Third, we controlled for diurnal variations in choroidal structure by measuring each participant at the same time of the day.

In summary, 3D CVI decreased gradually with increasing severity of myopia. FSV% in the CC was negatively correlated with 3D CVI and CT. These results may indicate that the choroidal vascularity of large- and middle-sized choroidal vessels decreases as myopia develops. CC blood perfusion showed a decreasing trend with worsening myopia and thinning of choroid. However, further longitudinal studies are needed on the complex relationship between choroidal vascularity and CC in the development and pathophysiology of myopia.

## Figures and Tables

**Table 1:** Demographical Characteristics of Included Individuals.

Characteristic	Low Myopia (n = 32)	Moderate Myopia (n = 36)	High Myopia (n = 45)	*p* Value
Age, y, median (IQR)	25.50 (8.00)	24.50 (13.75)	28.00 (10.00)	0.253 *
Female, n (%)	24 (66.67%)	28 (62.22%)	14 (46.7%)	0.667 ^†^
AL, mm, mean ± SD	23.78 ± 0.92	25.30 ± 0.97	26.55 ± 1.19	<0.001 ^‡^
Spherical equivalent, D, median (IQR)	−1.50 (1.44)	−4.75 (1.25)	−7.00 (1.88)	<0.001 *

IQR, interquartile range; AL, axial length; SD, standard deviation. * *p* value determined by Kruskal-Wallis H test; ^†^ *p* value determined by one-way ANOVA test; ^‡^ *p* value determined by Chi-square test.

**Table 2:** Multivariate regression analysis of central macular and global choroidal parameters with age and AL.

	Age			AL			
Variables	B	*β* Coefficient	*p* Value	B	*β* Coefficient	*p* Value	Adjusted R^2^
CT_C1 (μm)	−0.251	0.024	0.762	−42.265	−0.599	**<0.001**	0.365
CT_C3 (μm)	−0.439	−0.043	0.577	−39.782	−0.594	**<0.001**	0.365
TCV_C1 (mm^3^)	−0.000	−0.024	0.762	−0.033	−0.599	**<0.001**	0.365
TCV_C3 (mm^3^)	−0.003	−0.043	0.577	−0.281	−0.594	**<0.001**	0.365
LV_C1 (mm^3^)	−0.000	−0.070	0.370	−0.017	−0.586	**<0.001**	0.364
LV_C3 (mm^3^)	−0.003	−0.097	0.221	−0.121	−0.557	**<0.001**	0.341
SV_C1 (mm^3^)	−0.000	0.023	0.788	−0.016	−0.513	**<0.001**	0.202
SV_C3 (mm^3^)	0.000	0.002	0.978	−0.160	−0.560	**<0.001**	0.313
3D CVI_C1 (%)	−0.163	−0.161	0.055	−3.065	−0.459	**<0.001**	0.266
3D CVI_C3 (%)	−0.151	−0.195	**0.025**	−2.032	−0.397	**<0.001**	0.226
FSV%_C1 (%)	0.175	0.475	**<0.001**	0.056	0.023	0.787	0.231
FSV%_C3 (%)	0.155	0.467	**<0.001**	0.341	0.156	0.063	0.271

CT, choroidal thickness; TCV, total choroidal volume; LV, luminal volume; SV, stromal volume; 3D CVI, three-dimensional choroidal vascularity index; FSV, flow signal voids; AL, axis length. Bold font indicates statistical significance.

## Data Availability

The data from this study are available from the corresponding author upon request.

## Supplementary Materials

The following materials are available online at https://www.mdpi.com/article/10.3390/diagnostics11111948/s1, Table S1: global analysis of choroidal parameters between different myopia groups, Table S2: correlation analysis between 3D CVI and other choroidal parameters.
