# Supplementary material for: Quantitative Assessment of Three-Dimensional Choroidal Vascularity and Choriocapillaris Flow Signal Voids in Myopic Patients Using SS-OCTA
Source: Diagnostics (Basel). 2021 Oct 20;11(11):1948. doi: 10.3390/diagnostics11111948 (PMC8618547; doi:10.3390/diagnostics11111948)
Supplement: Supplementary file 1 [file diagnostics-11-01948-s001.zip › diagnostics-1389555-supplementary.pdf]

Table S1. Global analysis of choroidal parameters between different myopia groups

	Mean ± SD			*P*			
**Parameter**	**Low myopia (n=32)**	**Moderate myopia (n=36)**	**High myopia (n=45)**	**Among groups**	**Low myopia vs Moderate myopia**	**Moderate myopia vs High myopia**	**Low myopia vs High myopia**
CT _C3 (μm)	394.60 (146.30)	285.55 (114.96)	245.67 (100.08)	**<0.001***	**0.003***	**0.015***	**<0.001***
TCV_C3 (mm^3)	2.79 (1.03)	2.02 (0.81)	1.74 (0.71)	**<0.001***	**0.003***	**0.015***	**<0.001***
LV_C3 (mm^3)	0.82 (0.46)	0.63 (0.49)	0.39 (0.33)	**<0.001***	0.073	**0.007***	**<0.001***
SV_C3 (mm^3)	1.82 (0.41)	1.47 (0.45)	1.29 (0.46)	**<0.001***	**0.001***	**0.025***	**<0.001***
3D CVI_C3 (%)	30.34 ± 7.36	29.06 ± 7.15	23.79 ± 7.58	**0.001†**	1.000	**0.006†**	**0.001†**
FSV%_C3 (%)	13.14 (3.85)	13.24 (4.19)	15.44 (5.01)	**0.036***	1.000	0.348	**0.036***

*P value determined by Kruskal-Wallis H test; †P value determined by One-Way ANOVA test.

All data were presented as mean ± standard deviation or median (interquartile range). Bold font indicates statistical significance.

Table S2. Correlation analysis between 3D CVI and other choroidal parameters

3D CVI_C1 (%)			3D CVI_C3 (%)		
**Parameter**	**Correlation Coefficient**	***P* value**	**Parameter**	**Correlation Coefficient**	***P* value**
CT_C1 (μm)	0.718	**<0.001**	CT _C3 (μm)	0.714	**<0.001**
TCV_C1 (mm^3)	0.718	**<0.001**	TCV_C3 (mm^3)	0.714	**<0.001**
LV_C1 (mm^3)	0.899	**<0.001**	LV_C3 (mm^3)	0.897	**<0.001**
SV_C1 (mm^3)	0.421	**<0.001**	SV_C3 (mm^3)	0.513	**<0.001**
FSV%_C1 (%)	-0.274	**0.003**	FSV%_C3 (%)	-0.421	**<0.001**

All analyses were performed by Spearman correlation analysis.

Bold font indicates statistical significance.
